# Validating the accuracy of a novel virtual reality platform for determining implant orientation in simulated primary total hip replacement

**DOI:** 10.1177/20552076221141215

**Published:** 2022-12-08

**Authors:** Daniel Howgate, Michael Oliver, Julie Stebbins, Patrick Garfjeld Roberts, Ben Kendrick, Jonathan Rees, Stephen Taylor

**Affiliations:** 1 Nuffield Department of Orthopaedics Rheumatology and Musculoskeletal Sciences, The Botnar Research Centre, University of Oxford, Oxford, UK; 2 552380 NIHR Oxford Biomedical Research Centre, The Joint Research Office, Oxford, UK; 3 159064 Oxford University Hospitals NHS Foundation Trust Nuffield Orthopaedic Centre, Oxford, Oxfordshire, UK, London, UK; 4 Dinwoodie Charitable Company and Royal College of Surgeons of England Research Fellow, London, UK; 5 105594 The MRC Weatherall Institute of Molecular Medicine, Oxford, UK

**Keywords:** Total hip replacement, medical education, surgical training, surgical simulation, implant positioning, virtual reality

## Abstract

**Introduction:**

Accurate acetabular cup and femoral stem component orientation are critical for optimising patient outcomes, reducing complications and increasing component longevity following total hip replacement (THR). This study aimed to determine the accuracy of a novel virtual reality (VR) platform in assessing component orientation in a simulated THR model.

**Methods:**

The VR platform (HTC Vive Pro® system hardware) was compared against the validated Vicon® optical motion capture (MoCap) system. An acetabular cup and femoral stem were manually implanted across a range of orientations into pelvic and femur sawbones, respectively. Simultaneous readings of the acetabular cup operative anteversion (OA) and inclination (OI) and femoral stem alignment (FSA) and neck anteversion (FNA) were obtained from the VR and MoCap systems. Statistical analysis was performed using Pearson product-moment correlation coefficient (PPMCC) (Pearson’s r) and linear regression (R^2^).

**Results:**

A total of 55 readings were obtained for the acetabular cup and 68 for the femoral stem model. The mean average differences in OA, OI, FSA and FNA between the systems were 3.44°, −0.01°, 0.01° and −0.04°, respectively. Strong positive correlations were demonstrated between both systems in OA, OI, FSA and FNA, with Pearson’s r = 0.92, 0.94, 0.99 and 0.99, and adjusted R^2^ = 0.82, 0.9, 0.98 and 0.98, respectively.

**Conclusion:**

The novel VR platform is highly accurate and reliable in determining both acetabular cup and femoral stem component orientations in simulated THR models. This adaptable and cost-effective digital tracking platform may be modified for use in a range of simulated surgical training and educational purposes, particularly in orthopaedic surgery.

## Introduction and aims

Restoration of normal hip biomechanics is an important aspect of total hip replacement (THR) and has been associated with improved patient function, increased component longevity and reduced incidence of complications.^1–6^ Achieving this goal requires careful pre-operative radiographic analysis and component templating,^7^ combined with meticulous intra-operative surgical technique and judgement to prepare, and implant correctly sized components in the optimal position and orientation. Accurate positioning and orientation of the acetabular cup is required to restore the hip centre of rotation and optimise the functional range of movement, whereas accurate femoral stem positioning and orientation is critical to balance leg lengths and optimise both hip joint stability and range of movement. The orientation of an acetabular cup is commonly described by its inclination and version, which can be assessed anatomically, radiographically or operatively. The acetabular cup operative anteversion (OA) has been defined as the angle subtended between the longitudinal axis of the patient and the acetabular axis in the sagittal plane.^8^ The acetabular cup operative inclination (OI), or the abduction angle, is the angle subtended between the acetabular axis and the sagittal plane. Femoral neck anteversion (FNA) is the angle subtended between the posterior bicondylar axis of the distal femur and a line up the centre of the femoral neck in the axial plane.^9^ Femoral stem alignment (FSA) is the angle subtended between a line down the anatomical long axis of the femur and a second line starting from the tip of the femoral stem travelling up the midpoint of the stem. In conventional THR, the surgeon controls component position and orientation when implanting cemented and uncemented acetabular cups and cemented femoral stems. Surgeons typically have less control of femoral stem version when using uncemented prostheses as this is largely dictated by the patient’s proximal femoral anatomy.^6^ Robotics and navigation systems are emerging digital technologies which use computer guidance to assist surgeons in achieving their desired component positioning and orientation in both hip and knee replacement operations.^10,11^

Contemporary surgical training programmes have reduced volume and breadth of in-training surgical experience compared with previous generations.^12,13^ Given this reduction, there is a need for a focused, evidence-based and unified approach to surgical training to maximise opportunities for trainees to achieve operative competence prior to completion of training. An increasing number of reports are emerging of the application of virtual (VR) and augmented reality (AR) platforms in the field of surgical training, and specifically THR.^14–22^ However, the accuracy of VR systems in estimating object position and orientation has been reported in relatively few published studies.^16,23–25^ Traditional laboratory research methods for assessing acetabular and femoral component positioning include radiographic analysis and stereo photogrammetry.^26^ Both methods are time-consuming and do not provide real-time feedback on user performance. The purpose of this study is to determine the feasibility, accuracy and reliability of VR tracking for THR component orientation. To do this we developed an open-source novel VR platform called Aescular VR (https://github.com/Taylor-CCB-Group/AesculaVR). Aescular VR provides a framework that allows calibration and measurement of angle and distance between multiple tracked objects in a 3D space. It also allows real time recording of these values to facilitate data capture. This was used in assessing both acetabular cup and femoral stem component orientation in a THR model in comparison to a validated, highly accurate and precise optical motion capture (MoCap) system.^27,28^

## Methods

Research ethics committee approval was not required for conducting this study as it did not involve human subjects, or any form of randomisation, and the results are specific to the experimental settings in which they were conducted. The study was conducted with institutional approval within a recognised university teaching hospital gait laboratory, which is used for both clinical and research purposes.

### Measurement systems and outcome metrics

The two component orientation measurement systems used in this study were:

1. The Vicon^®^ optical MoCap system (16 × T-series cameras capturing at 100 Hz, Oxford Metrics PLC, Oxford, UK). The readings from this system were used as the ‘gold standard’ for comparison.
2. The VR platform (Aescular VR) developed using HTC Vive Pro^®^ system hardware including two base stations with stands (‘lighthouses’) and two HTC Vive trackers (HTC, New Taipei, Taiwan).

**Figure 1.**
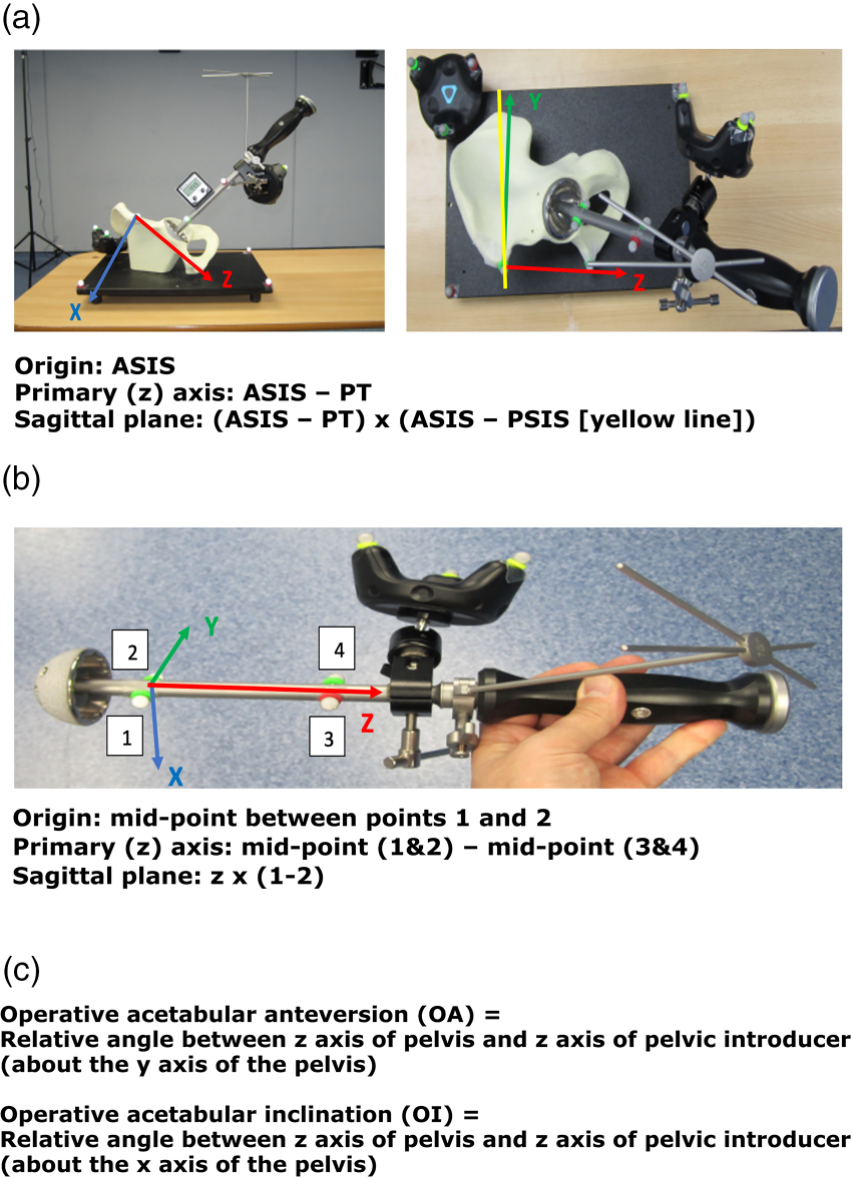
(a) Segment definition for the pelvis, (b) segment definition for the pelvis introducer and (c) angle definitions.

**Figure 2.**
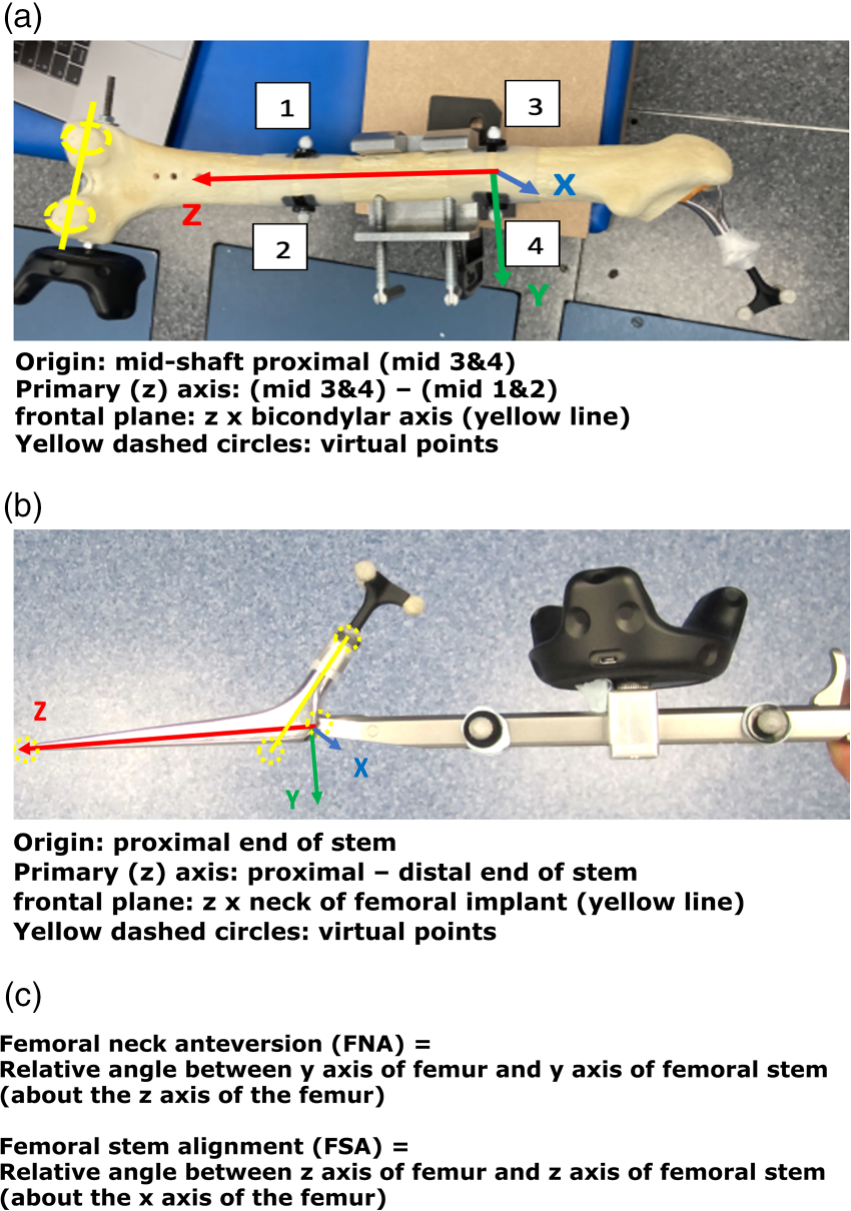
(a) Segment definition for the femur, (b) segment definition for the femoral stem and (c) angle definitions.

The prosthetic implants used were a 56mm uncemented acetabular cup in the pelvis model (Trinity™, Corin Group Ltd, The Corinium Centre, Cirencester, UK) and a size 1 collarless polished tapered stem in the femur model (Taperfit™, Corin Group Ltd, The Corinium Centre, Cirencester, UK). Following calibration of the MoCap and VR systems the acetabular and femoral prostheses were manually implanted in prepared synthetic adult male left hemi-pelvis and femur bone models (Sawbones^®^ Europe AB, Limhamn, Sweden) across a range of orientations representative of those expected intra-operatively.^29–31^ The synthetic hemi-pelvis and femur bone models were securely attached to level baseplates to simulate the most commonly used patient position and surgical approach for performing a THR, namely the lateral decubitus position (Figure 1) and posterior surgical approach (Figure 2).^32^ The acetabular cup was implanted within an intended range of OA between 10 and 40° at 5° increments and OI between 35 and 60° at 5° increments. A digital inclinometer was used as a reference for acetabular cup OI, and a mechanical alignment guide (MAG) as a reference for OA.^26,33,34^ The femoral stem was implanted freehand within an intended range of FNA between 0 and 30° at 5° increments and FSA of neutral (0°), maximal varus or maximal valgus. A modelling compound was used for simulated bone cement when inserting the femoral stem (Play-Doh, Hasbro, Pawtucket, Rhode Island, USA). This was injected into an appropriately prepared femoral canal using a high-pressure caulking gun with a long nozzle (Wolfcraft Gmbh, MG600 PRO, Kempenich, Germany). Following component implantation, simultaneous orientation measurements were taken using the MoCap and VR systems. The prostheses were removed and reimplanted for each individual experiment. The component orientation measurements collected for each experiment were the OI and OA on the model hemi-pelvis; and the FNA and FSA on the model femur.

### Protocol for data collection

The simulated synthetic bone model being tested was positioned in the centre of the gait laboratory, with full view of the MoCap cameras and VR lighthouses. Physical passive markers were placed on the pelvis and femur models, and on the THR components, to allow calculation of three-dimensional orientation by the MoCap system. The MoCap system requires line of sight for each marker. Where this was not physically possible (e.g. the distal end of the femoral stem once implanted) virtual points were set using a wand prior to implantation. The VR wireless trackers and passive optical motion markers from the MoCap system were positioned on the synthetic bone models and surgical instruments as follows (Figures 1 and 2):

VR tracker positions:

- Single tracker on the model baseplate (pelvis) or synthetic bone (femur).
- Single tracker on either the acetabular cup or femoral stem introducer handle.

Passive optical marker positions:

- Pelvis synthetic bone
  - Two markers placed over anterior superior iliac crest (ASIS) and pubic tubercle (PT) to define the anterior pelvic plane (reference for measuring OA).
  - Three markers over the baseplate to define the virtual sagittal plane (reference for measuring OI).
- Acetabular cup introducer
  - Four markers were placed on the introducer handle: anterior and posterior both proximal and distal.
- Femur synthetic bone
  - Two markers placed on medial/lateral femur 15cm distal to tip of greater trochanter and a further two markers placed 15cm distal to this (reference for measuring FSA).
  - Posterior condylar axis marked with wand (reference for measuring FNA).
  - Centre of the intercondylar notch marked with wand (reference for measuring FSA).
- Femoral stem and introducer
  - Four markers were placed on the introducer handle: anterior and posterior both proximal and distal to the VR tracker.
  - Tri-cluster marker at the apex of the trunnion.
  - The tip of stem was marked with the wand (reference for measuring FSA).
  - The lateral stem at intersection of neck axis (125°) was marked with the wand (reference for the NSA).

The segment and relative angle definitions used to calculate component orientation measurements for the acetabular cup and femoral stem are outlined in Figures 1 and 2, respectively. Calibration of these segment angles and planes using the VR and MoCap systems was performed prior to experimental data collection. This process should accommodate for any subsequent movement in position or orientation of either the synthetic bone or surgical instrument segments, as the pose of the VR trackers and passive optical motion markers remain fixed relative to each segment. This setup provides user flexibility in generating a range of simulated scenarios for each model, for example, adjusting pelvic tilt to recreate intra-operative pelvic adduction.^35^

### Statistical analysis

The Pearson product-moment correlation coefficient (PPMCC) was used to determine the strength of any relationship between the readings from the MoCap and the VR systems.^36^ Linear regression analysis was conducted to determine the coefficient of determination (R^2^) between readings from MoCap system and VR systems. Bland–Altman (BA) plots and statistics were used to assess the level of agreement between MoCap system and the VR systems. The significance threshold was set at 0.05 for all statistical analysis. Data analysis and visualisation was performed using RStudio (RStudio Team (2021): Integrated Development for RStudio, PBC, Boston, MA, USA).

## Results

### Pelvis model

A total of 55 experiments were conducted investigating the OA and OI using the MoCap and VR systems on the pelvis model. The mean difference in OA between the MoCap and VR system was 3.44°, 95% CI [−3.04 to 9.90°], *p* < 0.001; and −0.01°, 95% CI [−4.51 to 4.50°], *p* < 0.001 in OI. Very strong positive correlations were demonstrated between the MoCap and VR system in both OA (PPMCC = 0.92, 95% CI [0.86–0.95], *p* < 0.001) and OI (PPMCC = 0.94, 95% CI [0.9–0.97], *p* < 0.001). Linear regression modelling demonstrated an adjusted R^2^ of 0.82 for OA and 0.9 for OI when comparing the MoCap and VR systems.

### Femur model

**Figure 3.**
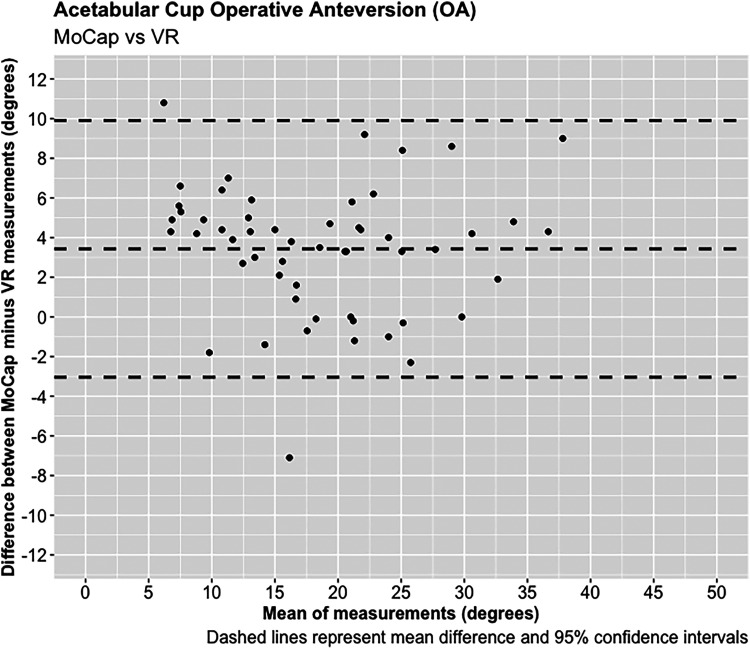
Acetabular cup operative anteversion (OA).

**Figure 4.**
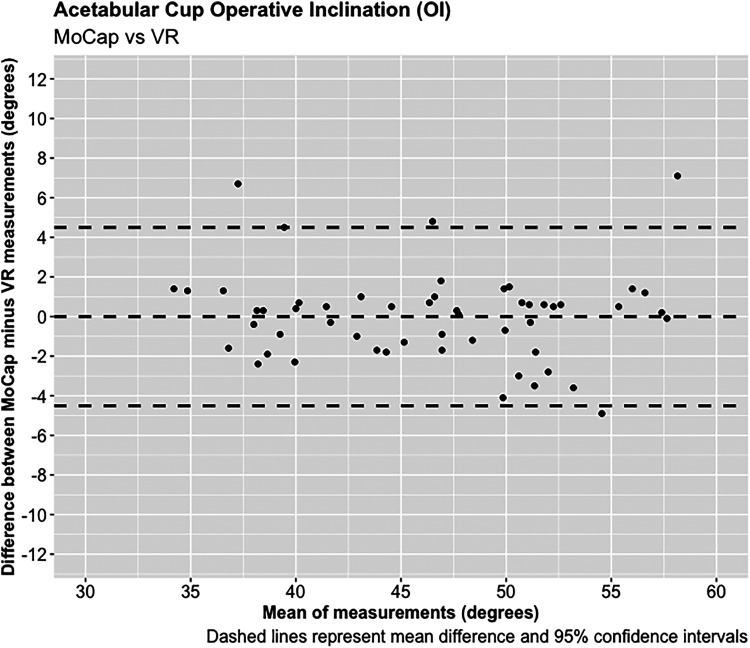
Acetabular cup operative inclination (OI).

**Figure 5.**
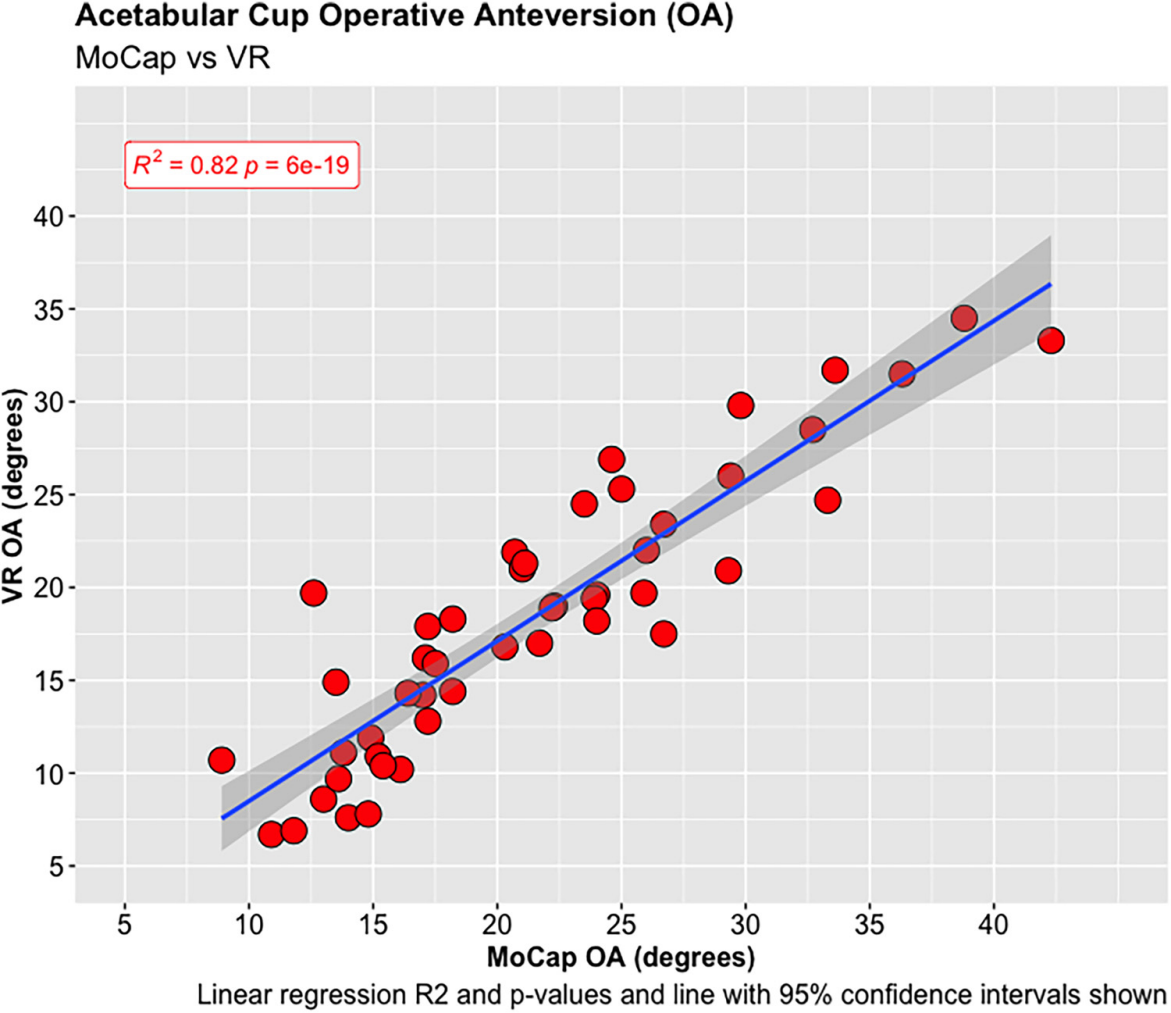
Acetabular cup operative anteversion (OA).

**Figure 6.**
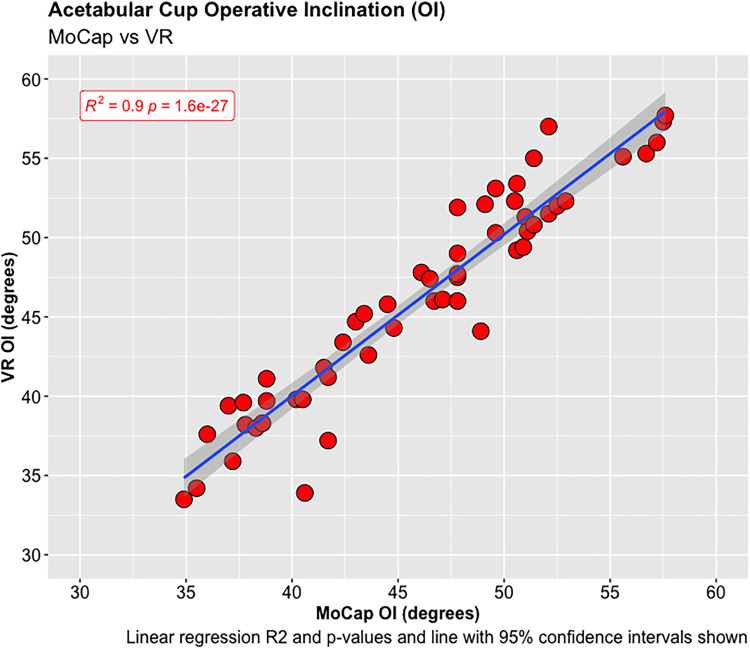
Acetabular cup operative inclination (OI).

**Figure 7.**
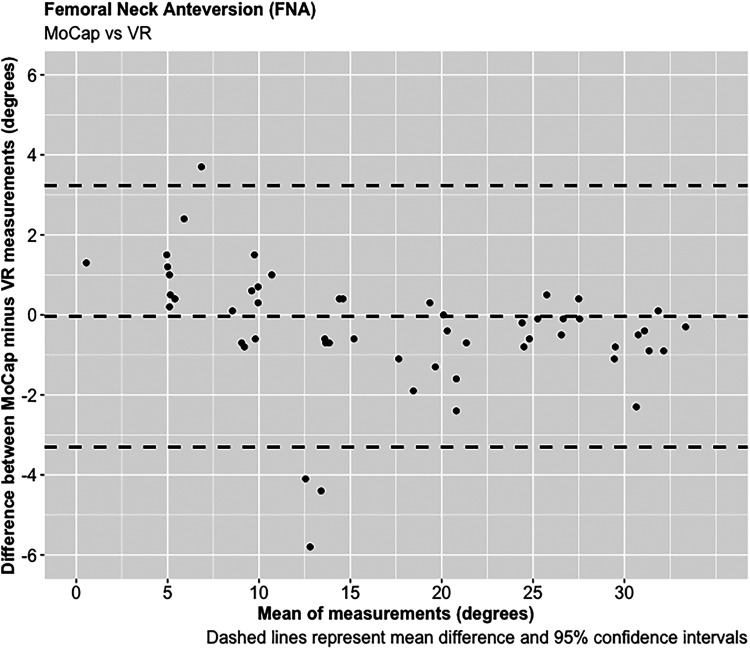
Femoral neck anteversion (FNA).

**Figure 8.**
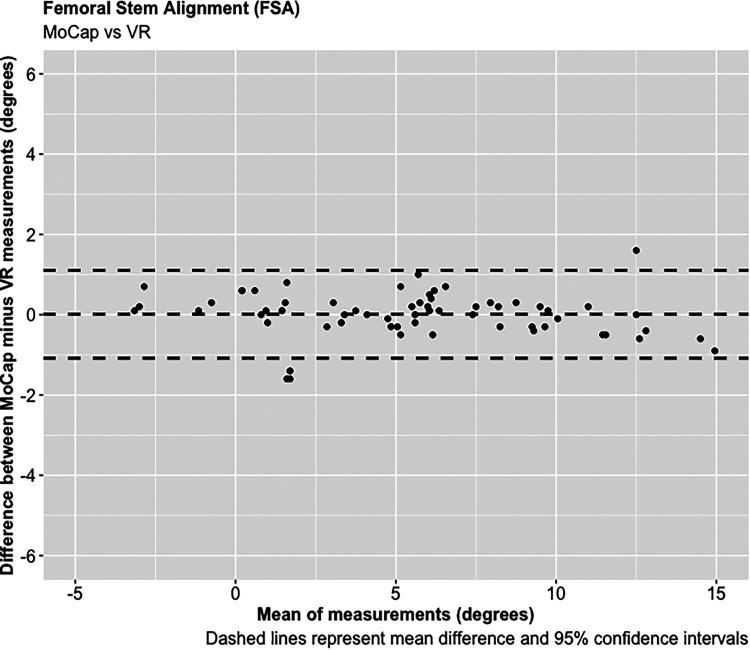
Femoral stem alignment (FSA).

**Figure 9.**
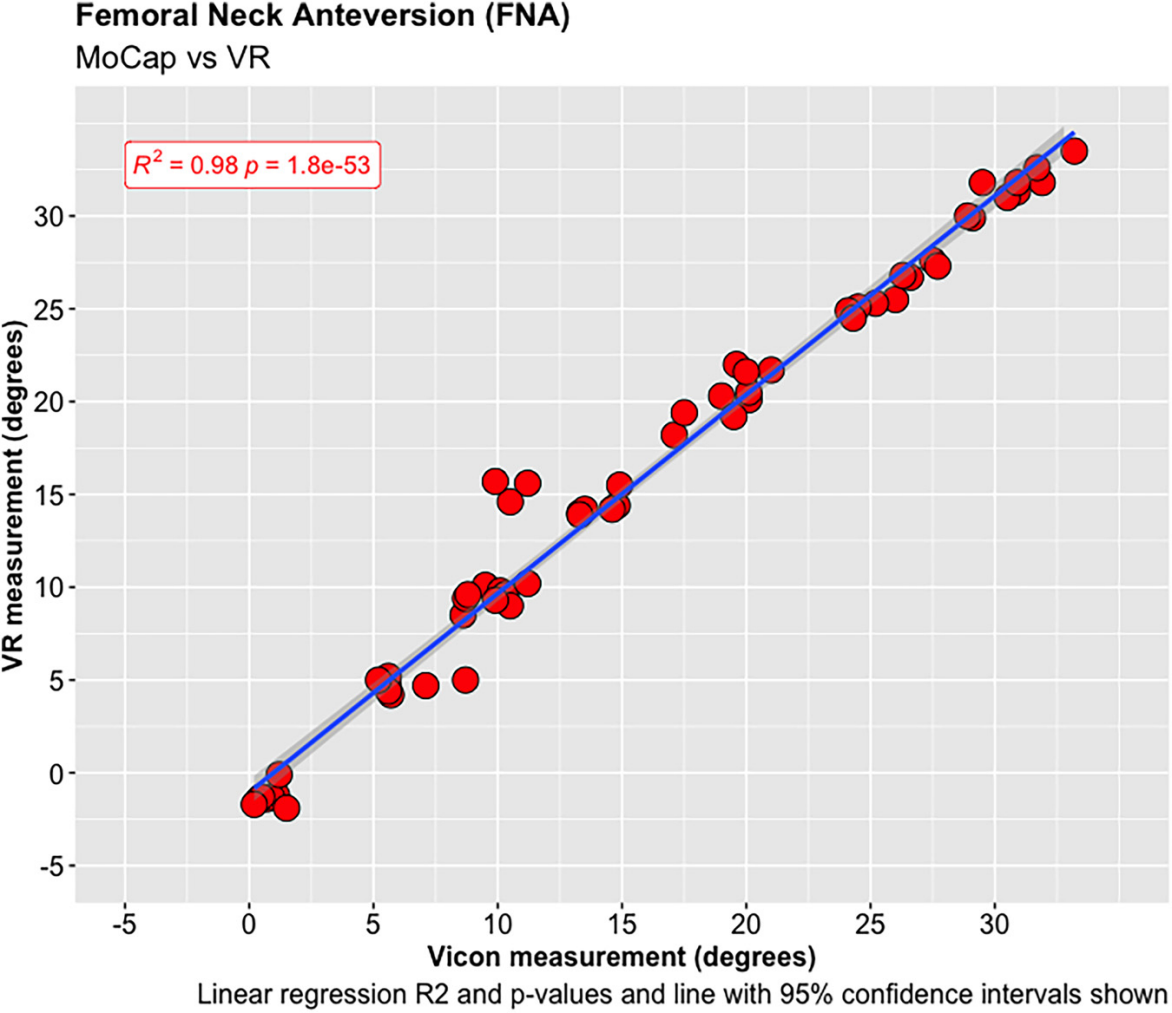
Femoral neck anteversion (FNA).

**Figure 10.**
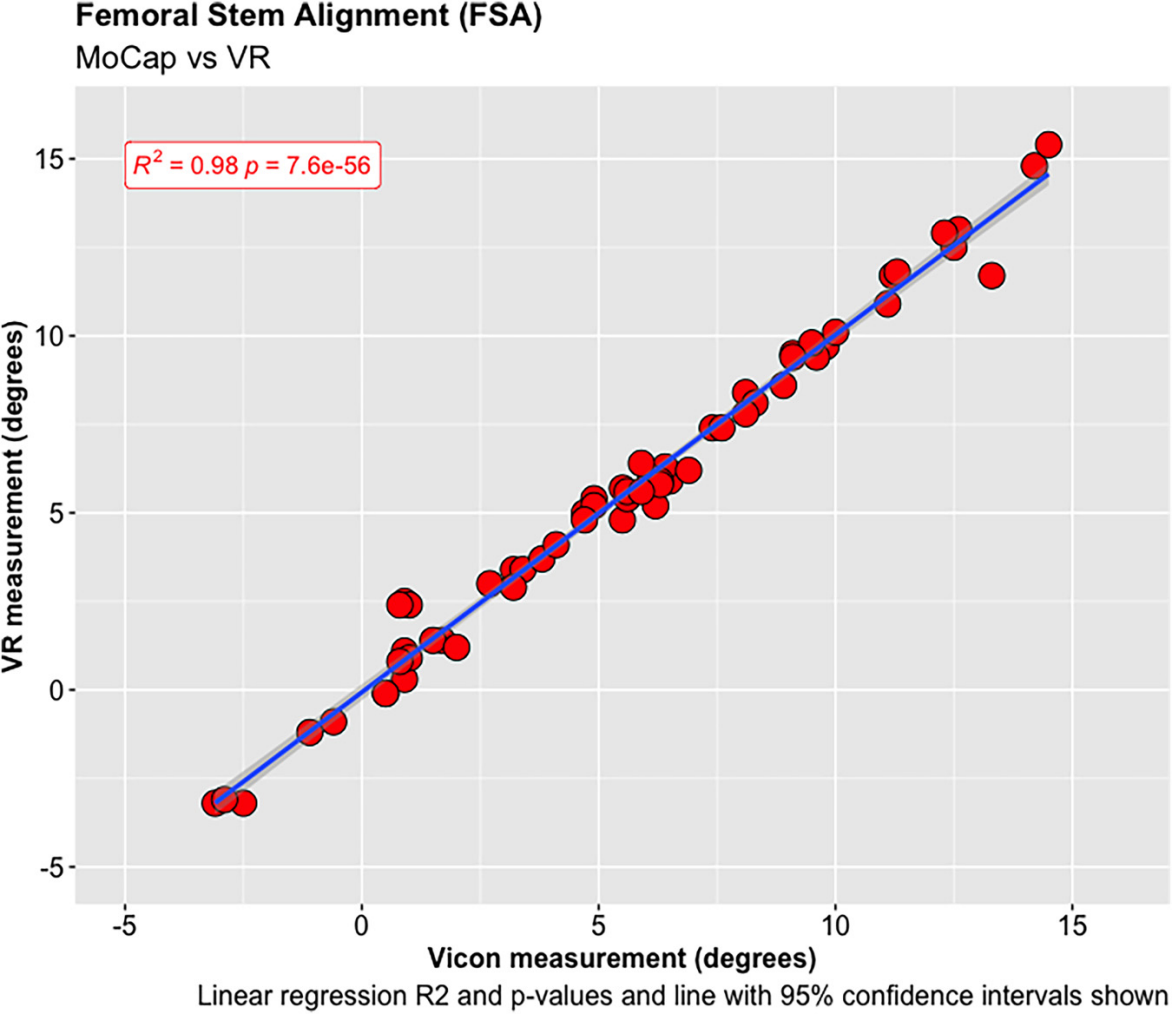
Femoral stem alignment (FSA).

A total of 68 experiments were conducted investigating the FNA and FSA readings using the MoCap and VR systems on the femur model. The mean average difference in FNA between the MoCap and VR system was −0.04°, 95% CI [−3.30 to 3.23°], *p* < 0.001; and 0.01°, 95% CI [−1.08 to 1.11°], *p* < 0.001 in FSA. Very strong positive correlations were demonstrated between the MoCap and VR system in both FNA (PPMCC = 0.99, 95% CI [0.98–0.99], *p* < 0.001) and FSA (PPMCC = 0.99, 95% CI [0.99–0.99], *p* < 0.001). Linear regression modelling demonstrated an adjusted R^2^ of 0.98 for both FNA and FSA when comparing the MoCap and VR systems (Figures 3–10) (Table 1)

**Table 1.** Summary of relationship and significance between the MoCap and VR system orientation measurements for the pelvis and femur models.

Model	Measurement	Pearson’s r	95% CI	*P*-value	Mean difference	Lower limit	Upper limit	Critical difference	Linear regression intercept	Linear regression coefficient
Pelvis	OA	0.92	0.86–0.95	< 2.2×10^−16^	3.44°	−3.04°	9.9°	6.47°	5.16	0.82
Pelvis	OI	0.94	0.9–0.97	< 2.2×10^−16^	−0.01°	−4.51°	4.5°	4.5°	3.91	0.9
Femur	FNA	0.99	0.98–0.99	< 2.2×10^−16^	−0.04°	−3.3°	3.23°	3.27°	1.24	0.98
Femur	FSA	0.99	0.99–0.99	< 2.2×10^−16^	0.01°	−1.08°	1.11°	1.09°	0.15	0.98

FSA: femoral stem alignment; FNA: femoral neck anteversion; MoCap: motion capture; OA: operative anteversion; OI: operative inclination; VR: virtual reality.

## Discussion

This study has investigated the development, application and validation of a novel and low-cost method using VR technology for determining real-time measurements of both acetabular cup and femoral stem component orientation in simulated THR. The benefit of using a live feedback mechanism for accurately determining component orientation is that it will allow surgical trainees to practise and optimise the psychomotor skills of THR in the safety of a simulated environment before translating these skills into the operating theatre. This system has already been successfully used within our institute as part of a simulation-based training module in THR and has received positive feedback.

VR trackers have been used for estimating object position and orientation in several applications to date.^16,23–25^ One study assessed the agreement between the HTC Vive^®^ VR system and the Vicon^®^ MoCap system when measuring lumbar postural changes, and reported a high degree of accuracy in position (0.68 + /−0.32cm) and orientation (1.64 + /−0.18°) estimation.^23^ Another study reported a high degree of accuracy in pose estimation for both static and multiplanar exercises.^25^ The results of our laboratory-based study indicate that the Aescular VR system tested has a mean average difference of −0.01° in OI and 3.44° in OA in comparison to the Vicon^®^ MoCap system, with very strong positive correlations between these systems in both OI and OA. The increased error observed between systems in determining acetabular cup OA in comparison to OI may relate to minor inaccuracies in defining the anterior pelvic plane during system calibration. This plane is used as the pelvic segment reference to calculate OA relative to the acetabular cup introducer segment which was more challenging to recreate virtually using the handheld VR trackers in comparison to the sagittal plane represented by the model baseplate from which OI is referenced (Figure 1). Although the accuracy of this VR system is higher in determining OI than OA, the 95% CIs for both measures fall within reported clinical acceptable margins of error for cup orientation of + /−10°,^30^ and therefore justify use of this system for assessing cup orientation in simulated THR.

To our knowledge, no published studies have reported using an AR or VR system for measuring femoral stem orientation in simulated THR, and this is a novel aspect of our research. We report a mean average difference of −0.04° in FNA and 0.01° in FSA between the Aescular VR and Vicon^®^ MoCap systems, with very strong positive correlations between these systems in both FNA and FSA. Anatomical studies report a mean average native FNA of 11.6° across both genders.^37^ For the majority of patients surgeons aim to achieve approximately 15° of FNA during THR, but a range between 10 and 20° is acceptable.^38^ The concept of combined anteversion between the acetabular cup OA and FNA, with a target range between 30 and 50° has also been reported.^29,39,40^ Clinical studies have reported that slight malalignment (0 + /−3°) of femoral stems are not associated with significant adverse patient outcomes following THR.^41,42^ The results of this feasibility study demonstrate that the precision of the VR system in determining FNA and FSA, as measured by the 95% CIs, falls within this acceptable range, and therefore justifies its use in simulated THR.

Studies have reported on the use and accuracy of AR-based systems for determining acetabular cup orientation in both simulated and clinical settings.^16,21,22^ An experimental AR system based on the Hololens™ headset (Microsoft^®^, Albuquerque, New Mexico, USA) was reported to demonstrate a translational accuracy of <1mm, with an orientation accuracy of 0.2° in inclination and 0.9° in anteversion during simulated THR.^16^ Another study investigated the accuracy of an AR system versus manual goniometer for determining acetabular cup orientation in a group of 54 patients undergoing primary THR via the direct anterior approach in a supine position.^21^ The authors report a mean absolute difference between the intra-operative AR and post-operative CT scan measurements of 2.1° + /−1.5° in inclination and 2.7° + /−1.7° in anteversion. A subsequent randomised controlled trial aimed to assess the accuracy of this AR system versus traditional MAGs in measuring acetabular cup orientation in a group of 46 patients undergoing primary THR via the Watson-Jones approach in a lateral decubitus position.^22^ The AR system in this study was calibrated using marker pins inserted into the iliac crests prior to moving patients from a supine into a lateral decubitus position. The mean difference between the AR system and post-operative CT scan measurements was 1.9° + /−1.3° in acetabular cup inclination, and 2.8° + /−2.2° in anteversion. Statistically significant, but not clinically important, differences were noted in acetabular cup inclination between systems, with conventional MAGs overestimating this measurement in comparison to the AR system. No significant differences were found between the AR system and MAG measurements in anteversion. The difference in inclination measurements may possibly be explained by the surgeon not fully accounting for intra-operative pelvic sag when using the MAG, which is a known factor for acetabular cup mal orientation in THR with patients in a lateral decubitus position.^35^ The main limitations of using this AR-based system for assessing acetabular cup orientation in the clinical setting are shared with established robotic-assisted navigation systems. These include the need for obtaining pre-operative CT scans, and often the intra-operative insertion of marker/array pins into bony reference points to allow system calibration, especially when patients are in a lateral decubitus position.

There are cost advantages to using a VR based system in surgical simulation training. The Vicon^®^ MoCap system used in our study costs approximately £200,000 GBP, in comparison to the VR system hardware which costs approximately £2100 (£1000 for a computer, £200 for two trackers, £900 for a Vive Pro Starter Kit which includes Headset, base station and controls). No cost analyses have been reported for the AR systems used for determining component orientation during simulated or clinical THR, and the software in these systems are currently not available on open-source platforms.^16,21,22^ The Aescular VR platform is also portable and can be set up and used in a variety of environments.^43^ A reduction in cost and improved access to VR technologies over the past decade has vastly increased their use and application for both research, commercial and recreational activities. It is estimated that AR and VR in the healthcare sector has a market value of approximately $2 billion USD in 2020, which is expected to increase further with future demand in this field.^44^ Medical and surgical training and education are only one application of this technology,^14–20^ with anatomy teaching also being a well cited use.^45–47^ Other reported applications of AR/VR technology in the healthcare setting include cognitive and physical rehabilitation following stroke or traumatic brain injury.^48–50^

The Aescular VR platform may be developed and applied in other domains for determining object pose estimation. The software is open source and can be modified to meet the requirements of users and intended application. This will hopefully allow other research groups to develop and validate novel applications of this technology quicker and with less effort. Further refinements to this software and calibration process may help to improve the accuracy and usability of this system.

## Conclusion

This study has demonstrated that the experimental VR platform tested is accurate in measuring both acetabular cup and femoral stem orientations in a laboratory setting using synthetic bone models. This system may be modified for use in a variety of surgical training and educational scenarios, particularly in orthopaedic surgery where accurate implant positioning and orientation are known to be critical to implant function, survivorship and patient outcomes.
